# Data on identification of primary and secondary metabolites in aqueous extract of *Verbascum betonicifolium*

**DOI:** 10.1016/j.dib.2020.106146

**Published:** 2020-08-06

**Authors:** Maria Luísa Serralheiro, Rita Guedes, Sezan R. Fadel, Hamdi Bendif

**Affiliations:** a BioISI - Biosystems & Integrative Sciences Institute, University of Lisbon, Faculty of Sciences, Lisboa, Campo Grande, 1749-016 Lisboa, Portugal; b Department of Chemistry and Biochemistry, University of Lisbon, Faculty of Science, Campo Grande, 1749-016 Lisboa, Portugal; c Technical University of Dresden, Bergstrasse 66, 01069 Dresden, Germany; d Natural and Life Sciences Department, Faculty of Sciences, University of M'sila, 28000 Msila, Algeria; e Laboratoire d'Ethnobotanique et des Substances Naturelles, Département des Sciences Naturelles, Ecole Normale Supérieure (ENS), Kouba, 16308 Alger, Algeria

**Keywords:** *Verbascum betonicifolium*, HRMS, Phytochemical composition, Secondary metabolite, Phenolic compounds, Terpenoids, Glycosidic derivatives

## Abstract

Tentative identification of primary and secondary metabolites in aqueous extracts from aerial parts of *Verbascum betonicifolium* Kuntze was done. This plant belongs to the Scrophulariaceae family and is used for several treatments in folk medicine. One of the processes commonly used to prepare this plant for consumption is boiling with water during approximately 20 minutes, that is, a decoction process. After filtration, this decoction was analysed in search for bioactive metabolites. The analysis was carried out by Electro-Spray Ionization (ESI) and High-Resolution Mass Spectrometry (HRMS) was done using a Quadropole Time-of-Flight (QToF, Impact II, Bruker), coupled to an Ultra High-Performance Liquid Chromatography (UHPLC, ELUTE autosampler, Bruker). The analysis was done in the negative mode (ESI-) and the identification was accomplished using the molecular formula suggestions from the Data Analysis 4.4™ software from Bruker and some databases, like Metlin and PubChem, always confirming with MS/MS results. These data can be used for finding biomarkers between *Verbascum* sps or to complementary medicine practitioners to get a scientific based knowledge of their results. These data are the unpublished supplementary materials related to “Bioactivities of Iridoids and flavonoids present in decoctions from aerial parts of *Verbascum betonicifolium*” (Fadel et al., 2020, submitted).

**Specifications Table**

**Table:** 

Subject	Analytical Chemistry
**Specific subject area**	Complementary and Alternative Medicines; Natural Products Metabolomics
**Type of data**	Figure Table
**How data were acquired**	The vial containing the aqueous extract stand in the autosampler of the ELUTE system, automatically injected into an Ultra High Performance Liquid Chromatography (UHPLC), and the eluted compounds from the chromatographic column were introduced into the electrospray ion source of a Quadrupole Time-of-Flight mass spectrometer (QToF Impact II, Bruker). The data was acquired using the Data Analysis 4.4™ software, from Bruker.
**Data format**	Raw (Figure) Secondary data: obtained from raw data (Table)
**Parameters for data collection**	Mass spectrometer conditions: ion spray voltage, –3.5 kV; nebulizer gas (N_2_), 2.0 bars; dry gas (N_2_), 4.0 L.min^−1^; dry heater, 200 °C; collision cell energy, 5.0 eV; end plate offset, 500 V. The internal calibration was performed using the high-precision calibration mode (HPC) and a solution that consisted of 250 mL H_2_O, 250 mL iPrOH, 750 μL acetic acid, 250 μL formic acid and 0.5 mL 1N NaOH solution, introduced to the ion source via a 20 μL loop before the sample enter the mass spectrometer, through a six-port valve. Data acquisition was performed in full scan positive mode in the range of m/z 50–1500, acquisition rate of 1 Hz. The Auto MS/MS mode was used to confirm the fragment ions. The liquid chromatography mass spectrometry (LC-MS) acquisition data were processed using the Data Analysis 4.4™ software, to extract the mass spectral features from samples raw data. Chromatograms with the retention times for several compounds leaving the chromatographic column, with different exact masses, could be obtained and each mass peak fragmentation could be searched for.
**Data source location**	Lisbon/Portugal/BioISI (mass spectrometry facility)/Universidade de Lisboa. Faculdade de Ciências/Latitude 38^o^45’26.09’’ N; Longitude 9^o^9’24.02’’W.
**Data accessibility**	http://dx.doi.org/10.17632/y4g36252cm.1
**Related research article**	Authors’ names Sezan R. Fadel^1^, Hamdi Bendif^2,3^, Laura Guedes^4^, Rebeca André^4^, Rita Pacheco^4,5^, Rita Guedes^4^, Karim Merabti^2^**,** Mohamed Djamel Miara^6^, Maria Luísa Serralheiro^4,7*^ Title Bioactivities of Iridoids and flavonoids present in decoctions from aerial parts of Verbascum betonicifolium (under evaluation)

## 1 Value of the data

• These data give the experimental conditions for separation and identification of leaves’ metabolites from plants aqueous extracts.
• Researchers working on plant metabolites and practitioners of natural medicines may have a scientific base for the results obtained.
• These data may be used by other researchers to compare secondary metabolites in plants of the same family, helping to find biomarkers between the species.

## 2 Data description

**Fig. 1:**
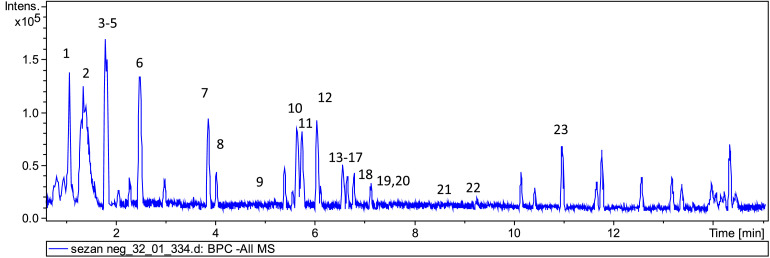
LC-MS chromatogram, in the negative mode, of the decoction from *Verbascum betonicifolium.*

Data from analysis by LC-MS/MS of the aqueous extract of the areal part of *Verbascum betonicifolium* prepared as decoction, in the negative mode, is shown in Fig 1.

## 3 Experimental design, materials and methods

[Offer a complete description of the experimental design and methods used to acquire these data. Please provide any programs or code files used for filtering and analyzing these data. It is very important

The 23 compounds indicated in Fig 1 were identified using the molecular formula suggested from the Data Analysis™ software and confirming by MS/MS analysis. The molecular formulas and the MS/MS fragments obtained for each proposed compound, as well its experimental mass and the respective error difference from the exact mass (ppm) are indicated in Table 1. Table 1 also has a tentative drawing of the structures to give an insight into its molecular design. These structures will help to explain any enzyme inhibitory activity and the antioxidant activity that may be detected with this extract when the enzyme inhibitory activity whose active site structure is known [1] and will help to propose through docking studies which will be the most appropriate inhibitor for the enzyme under evaluation.

**Table 1:** Tentative identification of compounds present in mucilage-free extract by High Resolution Mass Spectrometry (HRMS), using LC–MS/MS, in the ESI negative mode.

	RT (min)	[M-H]^−^	Formula	Error (ppm)	MS/MS product ions	Proposed compound	Putative structure
1	1.1	133.0144	C_4_H_6_O_5_	−1.3	115 (10 %); 72 (84.2 %); 71 (100 %); 59 (27.5 %)	malic acid	
2	1.3	195.0508	C_6_H_12_O_7_	1.1	89 (8.7 %); 87 (23 %); 85 (18.5 %); 75 (100 %); 72 (24 %); 71 (22.6 %); 59 (71.7 %); 57 (9.1%)	gluconic acid	
3	1.8	191.0194	C_6_H_8_O_7_	1.8	111 (77.3 %); 87 (100 %); 85 (30.7 %); 67 (7.1 %); 59 (15.2 %); 57 (28.1 %)	citric acid	
4	1.8	217.0352	C_8_H_10_O_7_	0.0	199 (2 %); 155 (6 %); 127 (13 %); 119 (8 %); 115 (30 %); 83 (100 %)	6-*O*-acetylascorbic acid	
5	1.9	407.1195	C_16_H_24_O_12_	0.0	181 (19 %); 163 (17 %); 151 (23 %); 113 (11 %)	unedide	
6	2.5	391.1245	C_16_H_24_O_11_	0.2	183 (23 %); 165 (26 %); 139 (14 %); 101(7%)	shanzhiside	
7	3.9	409.1344	C_16_H_26_O_12_	1.8	183 (100 %); 179 (25 %); 165 (44 %); 121(7%)	glycoside derivative^a^	
8	4.1	393.1401	C_16_H_26_O_11_	0.3	175 (19 %); 127 (21 %); 132 (3 %); 113 (4 %)	Methyl scutelloside	
9	5.1	353.0883	C_16_H_18_O_9_	−1.3	191 (100 %)	chlorogenic acid	
10	5.7	637.1028	C_27_H_26_O_18_	0.4	461 (40.3 %); 285 (100 %)	luteolin 7-glucuronosyl-(1-2) -glucuronide	
11	5.8	621.1091	C_27_H_26_O_17_	1.0	445 (41%); 269 (100%)	apigenin 7-glucuronosyl- (1-2)-glucuronside	
12	6.1	669.2031	C_30_H_38_O_17_	0.1	354 (1 %); 325 (3.4 %); 179 (8 %); 161 (55 %); 135 (31 %)	taxifolin derivative^b^	
13	6.5	623.1997	C_29_H_36_O_15_	−2.4	623 (40.5 %); 461 (11.7 %); 161 (100 %); 133 (33.7 %)	verbascoside	
14	6.6	447.0932	C_21_H_20_O_11_	0.2	447 (32.1 %); 285 (100 %); 284 (31 %); 227 (16 %); 201 (10.6 %); 133 (10.3 %); 107 (14.3 %)	luteolin 7-O-glucoside	
15	6.6	461.0722	C_21_H_18_O_12_	0.7	285 (100 %); 133 (16.6 %)	luteolin 7-O-glucuronide	
16	6.6	653.2082	C_30_H_38_O_16_	0.8	653 (100 %); 377 (39.4 %); 309 (11.3 %); 163 (74.6 %); 145 (89.6 %); 119 (52.5 %); 117 (23.7 %)	saccatoside	
17	6.7	401.1819	C_19_H_30_O_9_	1.0	221 (15 %); 195 (1 %); 162 (1 %); 71 (28 %); 59 (47 %)	sauroposide	
18	6.8	683.2154	C_31_H_40_O_17_	5.6	407 (41 %); 193 (60 %); 163 (11 %); 160 (65 %); 149 (9 %)	Scropheanosi de II	
19	7.1	431.0981	C_21_H_20_O_10_	0.7	-	apigenin 7-O-glucoside	
20	7.2	445.0770	C_21_H_18_O_11_	1.4	269 (100 %); 117 (16.3 %)	apigenin 7-galactoronide	
21	8.4	285.0398	C_15_H_10_O_6_	2.2	285 (100 %); 217 (9.1 %); 201 (6.7 %); 199 (11.3 %); 175 (10.8 %); 151 (15.9 %); 149 (7.3 %); 133 (41.4 %); 132 (12.4 %);	luteolin	
22	9.2	269.0456	C_15_H_10_O_5_	−0.2	269 (100 %); 227 (31.9 %); 165 (19.7%); 159 (6.9 %); 151 (70.6%); 149 (34.6 %); 117 (60.8 %); 107 (24.3 %);	apigenin	
23	11.7	293.1759	C_17_H_26_O_4_	0.2	236 (28 %); 221 (100 %)	phytuberin	

a : 1-[(2*R*,3*R*,4*S*,5*S*,6*R*)-2-[(3*S*,4*S*,5*R*)-3,4-dihydroxy-2,5-bis(hydroxymethyl)oxolan-2-yl]oxy-3,4,5-trihydroxy-6-(hydroxymethyl)-3-methyloxan-2-yl]prop-2-en-1-one

b : 5-Hydroxy-7-(2-hydroxyethoxy)-2-(3-hydroxy-4-methoxyphenyl)-3-[(2R,3S,4R,5R,6S)-3,4,5-trihydroxy-6-[[(2R,3R,4R,5R,6S)-3,4,5-trihydroxy-6-methyloxan-2-yl]oxymethyl]oxan-2-yl]oxy-2,3-dihydrochromen-4-one

## 4 Experimental design, materials and methods

The plant was collected in Algeria and prepared according to the process described in [1]. After filtration of the decoction, the aqueous extract was lyophilized, and a powder was obtained. For LC-MS/MS analysis, 1 mg was dissolved in MiliQ water (Millipore) and the vial was introduced in the autosampler. A chromatographic column Intensity Solo 2 RP-18, 100 × 2.1 mm, 2.0 µm column (Bruker, Bremen, Germany) was used. The elution conditions were described in [1], briefly: 5 µL were injected (auto injector) and a flow rate of 0.250 mL/min for the elution was used. The column was kept at 35°C and the samples at 10°C. The eluting gradient was composed of gradient of water with 0.1% formic acid (eluent A) and acetonitrile with 0.1% formic acid (eluent B) as follows: 0 min – 95% A; 1.5 min – 95% A; 13.5 min – 25% A; 18.5 min – 0% A; 21.5 min – 0% A; 23.5 min – 95% A; 30 min – 95% A. The chromatographic equipment was an ELUTE autosampler (UHPLC) from Bruker (Bremen, Germany) coupled to an Ultra-High-Resolution Quadrupole Time-of-Flight mass spectrometer (UHR-QToF, Impact II) also from Bruker (Bremen, Germany). The mass analysis was carried out with the parameters indicated in [2], briefly: the mass spectrometer was operated in the negative mode with the following parameters: ion spray voltage, –3.5 kV; nebulizer gas (N_2_), 2.0 bars; dry gas (N_2_), 8.0 L.min^−1^; dry heater, 200 °C; collision cell energy, 5.0 eV; end plate offset, 500 V. Calibration of masses was done by internal calibration method using a solution that consisted of 250 mL H_2_O, 250 mL iPrOH, 750 μL acetic acid, 250 μL formic acid and 0.5 mL 1N NaOH solution, on the HPC mode. The calibration solution was introduced into the ion source via a 20 μL loop, before the sample enter the mass spectrometer, through a six-port valve. The acquisition was performed in full scan mode in the 50–1500 m/z range, with an acquisition rate of 1 Hz. The Auto MS/MS mode was used to confirm the fragment ions. The LC-MS acquired data were processed using Data Analysis 4.4™ software (Bruker) to extract the mass spectral features from the sample raw data. The suggestion of chemical formulas according to the exact mass was done using the Data Analysis™ software. These suggestions were verified using online search in the databases Metlin (https://metlin.scripps.edu/landing_page.php?pgcontent=mainPage), in the simple search mode, and PubChem (https://pubchem.ncbi.nlm.nih.gov/) and confirmed through MS/MS analyses. For this analysis, the «Fragmentation Explorer» from Data Analysis™ software was used.

## Declaration of Competing Interest

The authors declare that they have no known competing financial interests or personal relationships which have, or could be perceived to have, influenced the work reported in this article.
